# Metabolic and Structural Consequences of GM3 Synthase Deficiency: Insights from an HEK293-T Knockout Model

**DOI:** 10.3390/biomedicines13040843

**Published:** 2025-04-01

**Authors:** Elena Chiricozzi, Giulia Lunghi, Manuela Valsecchi, Emma Veronica Carsana, Rosaria Bassi, Erika Di Biase, Dorina Dobi, Maria Grazia Ciampa, Laura Mauri, Massimo Aureli, Kei-ichiro Inamori, Jin-ichi Inokuchi, Sandro Sonnino, Maria Fazzari

**Affiliations:** 1 Department of Medical Biotechnology and Translational Medicine, Università Degli Studi di Milano, 20054 Segrate, Italy; giulia.lunghi@unimi.it (G.L.); manuela.valsecchi@unimi.it (M.V.); emma_sn@hotmail.it (E.V.C.); rosaria.bassi@unimi.it (R.B.); dorina.dobi@unimi.it (D.D.); maria.ciampa@unimi.it (M.G.C.); laura.mauri@unimi.it (L.M.); massimo.aureli@unimi.it (M.A.); sandro.sonnino@unimi.it (S.S.); 2 The Broad Institute of MIT and Harvard, Cambridge, MA 02142, USA; edibiase@broadinstitute.org; 3 Division of Glycopathology, Institute of Molecular Biomembrane and Glycobiology, Tohoku Medical and Pharmaceutical University, Sendai 981-8558, Japan; kinamori@tohoku-mpu.ac.jp; 4 Forefront Research Center, Graduate School of Science, Osaka University, Toyonaka 565-0871, Japan; inokuchi@chem.sci.osaka-u.ac.jp

**Keywords:** GM3 synthase deficiency, gangliosides, neurodegeneration, lysosomes, plasma membrane

## Abstract

**Background**: GM3 Synthase Deficiency (GM3SD) is a rare autosomal recessive neurodevelopmental disease characterized by recurrent seizures and neurological deficits. The disorder stems from mutations in the *ST3GAL5* gene, encoding GM3 synthase (GM3S), a key enzyme in ganglioside biosynthesis. While enzyme deficiencies affecting ganglioside catabolism are well-documented, the consequences of impaired ganglioside biosynthesis remain less explored. **Methods**: To investigate GM3SD, we used a Human Embryonic Kidney 293-T (HEK293-T) knockout (KO) cell model generated via CRISPR/Cas9 technology. Lipid composition was assessed via high-performance thin-layer chromatography (HPTLC); glycohydrolase activity in lysosomal and plasma membrane (PM) fractions was enzymatically analyzed. Lysosomal homeostasis was evaluated through protein content analysis and immunofluorescence, and cellular bioenergetics was measured using a luminescence-based assay. **Results**: Lipidome profiling revealed a significant accumulation of lactosylceramide (LacCer), the substrate of GM3S, along with increased levels of monosialyl-globoside Gb5 (MSGb5), indicating a metabolic shift in glycosphingolipid biosynthesis. Lipid raft analysis revealed elevated cholesterol levels, which may impair microdomain fluidity and signal transduction. Furthermore, altered activity of lysosomal and plasma membrane (PM)-associated glycohydrolases suggests secondary deregulation of glycosphingolipid metabolism, potentially contributing to abnormal lipid patterns. In addition, we observed increased lysosomal mass, indicating potential lysosomal homeostasis dysregulation. Finally, decreased adenosine triphosphate (ATP) levels point to impaired cellular bioenergetics, emphasizing the metabolic consequences of GM3SD. **Conclusions**: Together, these findings provide novel insights into the molecular alterations associated with GM3SD and establish the HEK293-T KO model as a promising platform for evaluating potential therapeutic strategies.

## 1. Introduction

Gangliosides are a class of glycosphingolipids essential to the nervous system, particularly within neuronal membranes [1,2]. Structurally, gangliosides consist of a ceramide backbone linked to a carbohydrate chain that includes one or more sialic acid residues [1]. These molecules are abundant in the outer layer of the plasma membrane (PM) and are key players in cell-to-cell communication, neuronal differentiation, synaptic plasticity, and modulation of signal transduction pathways [1,2].

On the PM, gangliosides drive the formation of “lipid rafts”, which are specialized microdomains composed of cholesterol, sphingolipids, dipalmitoylphosphatidylcholine, and specific proteins in lower quantities [3]. Lipid rafts are crucial for organizing ganglioside–receptor interactions and for modulating cellular responses to external stimuli [3,4]. In neurons, lipid rafts are involved in processes such as neurotransmission, axon guidance, and growth factor signaling, acting as centers for clustering signaling molecules and enhancing signal transduction efficiency [2,3].

Ganglioside’s biosynthesis occurs in the Golgi apparatus, where ceramide serves as a precursor [1,5,6]. Glycosyltransferases sequentially add monosaccharides and sialic acid residues to form different ganglioside species [6,7,8]. Degradation of gangliosides takes place in lysosomes, where they are broken down by lysosomal hydrolases into simpler molecules [5,9]. Alterations in this tightly regulated process can lead to the accumulation of undegraded glycosphingolipids, a hallmark of lysosomal storage disorders such as Tay–Sachs and Gaucher diseases [6].

Besides diseases caused by aberrant gangliosides catabolism, the pathological consequences of alterations in biosynthetic enzymes, including GM3 synthase (GM3S), have recently emerged [10]. GM3S is encoded by the *ST3GAL5* gene and uses lactosylceramide (LacCer) as a substrate to produce GM3, the precursor of a- and b-series gangliosides [1,2,5,6]. Biallelic mutations in the *ST3GAL5* gene lead to GM3 Synthase Deficiency (GM3SD), a disorder characterized by abnormal pigmentation, profound developmental delay, somatic growth failure, progressive microcephaly, intellectual disability, intractable seizures, deafness, and decreased survival [11,12,13,14,15,16,17]. The pathology was first described in 2004 within the Old Order Amish community [11] but subsequently also in non-Amish populations [12,13,14,15,16]. Individuals lacking GM3S appear normal at birth, but throughout the first six to twelve months of life they start to exhibit neurological deterioration [17]. Since the cellular and molecular consequences of GM3SD are still poorly understood, to date, no cure is available for GM3SD, and symptoms, especially seizures, are often untreatable. Additionally, although studying human GM3SD has been aided by mice carrying ganglioside synthesis defects, the available models display neurological impairments that are milder than those observed in patients [16,18].

This study aimed to characterize the consequences of GM3SD in a readily accessible human-derived in vitro model, Human Embryonic Kidney 293-T *ST3GAL5* knockout (KO) cells [19], with respect to wild-type (WT) cells. The investigation focused on highlighting the lipid profile, assessing the activities of enzymes involved in lipid synthesis, and exploring how the primary deficiency in GM3S leads to secondary alterations, not only in the lipid environment but also in other cellular processes. Our results reveal a general accumulation of LacCer and increased production of monosialyl-globoside Gb5 (MSGb5) in *ST3GAL5* KO cells. Intracellularly, we observed altered activity of glycosphingolipid catabolic enzymes and enhanced lysosome biogenesis. At the cell surface, glycosphingolipid hydrolases were notably deregulated, accompanied by elevated cholesterol levels in lipid rafts. These findings suggest that GM3SD induces significant lipid remodeling and a secondary glycosphingolipid metabolic alteration in both lysosomes and PMs, potentially affecting PM structure and function. Additionally, the increase in cholesterol within lipid rafts may reduce microdomains’ fluidity, potentially impairing signal transduction. Finally, we detected a decrease in adenosine triphosphate (ATP) production, indicating an underlying energetic deficiency.

## 2. Materials and Methods

### 2.1. Materials

The commercial chemicals used in this study were of the highest purity available; common solvents were distilled before use, and water was doubly distilled in a glass apparatus. Phosphate-buffered saline (PBS), paraformaldehyde, sucrose, sodium orthovanadate (Na_3_VO_4_), sodium chloride (NaCl), dithiothreitol, bovine serum albumin (BSA), phenylmethanesulfonyl fluoride (PMSF), protease inhibitor cocktail, aprotinin, Triton X-100 (TX-100), ethylenediaminetetraacetic acid (EDTA), sodium dodecyl sulfate (SDS), glycine, poly-L-lysine, methanol, triethylamine, chloroform, propanol, glycerol, Trizma base, citric acid, Na_2_HPO_4_, *Vibrio cholerae* sialidase, blue bromophenol and Dulbecco’s Modified Eagle Medium (DMEM), Conduritol B epoxide, AMP-Deoxynojirimycin, and high-performance thin-layer chromatography (HPTLC) plates were obtained from Merck Millipore (St. Louis, MO, USA). L-Glutamine, penicillin/streptomycin solution, and Fetal Bovine Serum were obtained from EuroClone (Paignton, UK). Hoechst solution, Trypsin/EDTA, Fluoromount-G reagent™ Mounting Medium, and DMEM-F12 without phenol red were obtained from Thermo Fisher Scientific (Waltham, MA, USA). A set of 4–20% Mini-PROTEAN^®^ TGX™ Precast Protein Gels, Turbo Polyvinylidene Difluoride (PVDF) Mini-Midi membranes, and a DC™ protein assay kit were obtained from Bio-Rad (Hercules, CA, USA). A Chemiluminescent kit for WB was obtained from Cyanagen (Bologna, Italy). Ultima gold and Black 96-well OptiPlate-96 F plates were obtained from Perkin Elmer (Waltham, MA, USA). Cell culture plates were obtained from Falcon Corning (Marlboro, NY, USA). 4-methylumbelliferyl β-D-glucopyranoside (MUB-β-Glc), 4-methylumbelliferyl β-D-galactopyranoside (MUB-β-Gal), 4-methylumbelliferyl α-D-galactopyranoside (MUB-α-Gal), 4-methylumbelliferyl N-acetyl-β-D-glucosaminide (MUG), 4-methylumbelliferyl N-acetyl-β-D-glucosaminide-6-sulfate (MUGS), and 4-methylumbelliferyl-β-D-mannopyranoside (MUB-β-Man) were all obtained from Glycosynth (Warrington, UK). 6-hexadecanoylamino 4-MU-phosphoryl-choline (HMUB-PC) was obtained from Moscerdam Substrates (Oegstgeest, The Netherlands).

### 2.2. Antibodies

For Western blotting (WB) and immunoblotting analyses, the following antibodies were used: rabbit polyclonal anti-translocase of outer mitochondrial membrane 20 [TOM20, research resource identifier number (RRID): AB_2207530] was purchased from ProteinTech (Manchester, UK); mouse anti-oxidative phosphorylation complexes (Oxphos) cocktail (RRID: AB_2629281) and rabbit polyclonal anti-flotillin (RRID: AB_941621) were obtained from Abcam (Cambridge, UK); rabbit polyclonal anti-glyceraldehyde-3-phosphate dehydrogenase (GADPH, RRID: AB_796208) was obtained from Merck Millipore (Burlington, MA, USA); mouse monoclonal anti-lysosomal associated membrane protein 1 (Lamp-1, H4A3; RRID: not available) was obtained from Developmental Studies Hybridoma Bank (Iowa City, IA, USA); rabbit polyclonal anti-microtubule-associated proteins 1A/1B light chain 3A (LC3, RRID: AB_796155) were obtained from Sig-ma-Aldrich (St. Louis, MO, USA); rabbit polyclonal anti-ubiquitin-binding protein p62 (SQSTM1/p62, RRID: AB_10624872), mouse polyclonal anti-Src Family Tyrosine Kinase Fyn (RRID: AB_10698604), and secondary horseradish peroxidase (HRP)-conjugated anti-rabbit IgG (RRID: AB_2099233) were obtained from Cell Signaling Technology (Danvers, MA, USA); mouse monoclonal anti-calnexin antibody (RRID: AB 397883) was obtained from BD Biosciences (San Jose, CA, USA); mouse monoclonal anti-prion protein (Prp, SAF32; RRID: not available) was obtained from Cayman Chemical (Ann Arbor, MI, USA); secondary HRP-conjugated goat anti-mouse IgG (H + L) antibody (RRID: AB_228309) was obtained from Life Technologies (Carlsbad, CA, USA).

### 2.3. Cell Culture

HEK293-T cells were obtained from the RIKEN cell bank (RCB2202) and cultured as monolayers in DMEM high-glucose medium supplemented with 10% heat-inactivated Fetal Bovine Serum, 1% L-glutamine, and 1% penicillin/streptomycin at 37 °C in a humidified atmosphere of 95% air/5% CO_2_. Cells were sub-cultured when growth reached 80–90% confluence (i.e., every 3–4 days). HEK293-T cells were plated at 30 × 10^3^/cm^2^ or 20 × 10^3^/cm^2^ and incubated to allow the generation of a monolayer (about 80% confluence) before treatments and/or analyses.

HEK293-T cells lacking GM3S were obtained, targeting the coding sequence for the sialyl L motif of the ST3GAL5 gene via the CRISPR/Cas9 technique, as previously described [20]. Briefly, a single exon of the human ST3GAL5 gene, containing the coding sequence for sialyl motif L, was selected for CRISPR guide RNA design using an online tool. The guide oligos (5′-CACCGCAAGACCTGTCGGCGCTGTG-3′ and 5′-AAACCACAGCGCCGACAGGTCTTGC-3′) were annealed and inserted into the pSpCas9(BB) plasmid (Addgene, Cambridge, MA, USA). The plasmid was then transfected into HEK293T cells using Lipofectamine 2000 (Thermo Fisher), following the manufacturer’s instructions. Clonal cell lines were obtained through single-cell cloning, and ganglioside expression levels were evaluated by TLC. 

### 2.4. Cell Culture Starvation

HEK293-T cells, plated and incubated for 72 h, were amino acid-starved in starvation medium (140 mM NaCl, 1 mM CaCl_2_, 1 mM MgCl_2_, 5 mM glucose, and 20 mM Hepes (pH 7.4)) for 3 h and 6 h at 37 °C in a humidified atmosphere of 95% air/5% CO_2_ at 37 °C. Control cells were supplemented with fresh complete culture medium containing 10% Fetal Bovine Serum.

### 2.5. Labeling of Cell Sphingolipids with [1-^3^H]-Sphingosine

A quantity of 36 nM [1-^3^H]-sphingosine (specific radioactivity: 1.36 µCi/mM) was administered to HEK293-T cells (70% confluence) to metabolically label cell sphingolipids, as previously reported [21]. [1-^3^H]-sphingosine solubilized in methanol was transferred into a sterile glass tube, dried under a nitrogen stream, and then dissolved in a proper volume of medium to a final concentration of 36 nM. The radioactivity linked to an aliquot of medium was evaluated by a beta-counter (PerkinElmer, Waltham, MA, USA), confirming the proper solubilization. Medium containing [1-^3^H]-sphingosine was administered to cells seeded 24 h before. Following a 24 h incubation period, the medium was discarded, and the cells were then cultured in a fresh culture medium free of radioactive sphingosine.

### 2.6. Protein Content Determination

In accordance with the manufacturer’s instructions, the DC™ protein assay kit (Bio-Rad, Hercules, CA, USA) was utilized to determine the protein concentrations of the samples, utilizing BSA at different concentrations as a standard.

### 2.7. Protein Analysis

HEK293-T cells were rinsed with cold PBS containing 1 mM Na_3_VO_4_, lysed with 80 μL/well (6-well plates) of Laemmli buffer (0.15 M dithiothreitol; 94 mM Tris-HCl; 15% glycerol, *v*/*v*; 3% SDS, *w*/*v*; 0.015% blue bromophenol, *v*/*v*), and sonicated with a Vibra-cell^TM^ (Sonics & Materials, Inc., Danbury, CT, USA) 3 times (10 s at 30% amplitude). After 5 min of boiling at 99 °C, equal amounts of protein were separated on a 4–20% precast gel or 15% acrylamide gels (for autophagy evaluation) and then transferred to PVDF membranes using the Trans-Blot^®^ Turbo™ Transfer System (Bio-Rad). Boiling lysates were avoided for optimal Oxphos subunit identification. PVDF membranes were incubated for 1 h in blocking solution [Tris-buffered saline containing 0.1% Tween-20 (TBS-T) and 5% non-fat dry milk] and then incubated overnight at 4 °C with the following primary antibodies: anti-TOM20 (1:1000 in 5% milk in TBS-T), anti-Oxphos subunits (1:2000 in 5% BSA in TBS-T), anti-Calnexin (1:1000 in 5% BSA in TBS-T), anti-GAPDH (1:5000 in 5% milk in TBS-T), anti-Flotillin (1:1000 in 5% milk in TBS-T), anti-Fyn (1:1000 in 5% milk in TBS-T), anti-Prp (1:2500 in 5% milk in TBS-T), anti-LC3 (1:1000 in 5% milk in TBS-T), anti-SQSTM1-p62 (1:1000 in 5% milk in TBS-T), and anti-Lamp-1 (1:100 in 5% milk in TBS-T). Using the proper HRP-conjugated secondary antibody (1:2000 in 5% milk in TBS-T or 5% BSA in TBS-T), blots were incubated for 1 h at 23 °C following 3 TBS-T washes. The Uvitec system (Cleaver Scientific Ltd., Rugby, UK) and the enhanced luminol-based chemiluminescent substrate were then used to visualize the immunocomplexes. Data were presented as a percentage of the WT (mean value of the WT set to 1.0), and band quantifications were performed using the ImageJ software (2.14.0; Java 1.8.0_322, NIH, Bethesda, MD, USA; http://rsbweb.nih.gov/ij/ accessed on 1 July 2023) [22].

### 2.8. Isolation of Detergent-Resistant Membrane (DRM) Fractions

Twenty-four hours after removing 36 nM [1-^3^H]-sphingosine from the cell culture medium, DRMs were prepared by ultracentrifugation on a discontinuous sucrose gradient of cells subjected to homogenization with 1% TX-100, as previously described [21,23,24]. Briefly, cells were mechanically collected in 1X PBS and centrifuged for 10 min at 270× *g*, 4 °C. The cell pellet was lysed in 1.2 mL of 1% TX-100 in 19 mM TNEV buffer (10 mM TrisHCl, 150 mM NaCl, and 5 mM EDTA (pH 7.5)) supplemented with 1 mM Na_3_VO_4_, 1 mM PMSF, 75 mU/mL aprotinin, and a protease inhibitor cocktail and then homogenized on ice 11 times using tight Dounce. The post-nuclear supernatant was obtained by centrifuging the cell lysate (2.5 mg of cell protein/mL) at 1300× *g* for 5 min at 4 °C to eliminate nuclei and cellular debris. A total of 1 mL of post-nuclear supernatant was mixed with an equal volume of 85% sucrose (*w*/*v*) in TNEV buffer containing 1 mM Na_3_VO_4_, loaded at the bottom of a discontinuous sucrose gradient (30–5%), and centrifuged for 17 h at 200,000× *g* at 4 °C. After ultracentrifugation, 12 fractions were collected, starting from the top of the tube. The light-scattering band, which represents the DRM, was situated at the interface between 5 and 30% sucrose, corresponding to fractions 5 and 6, while fractions 10, 11, and 12 comprised high-density (HD) fractions. The entire process was performed while submerged in ice at 0–4 °C. The radioactivity associated with the gradient fractions, as well as the quantity of fraction-specific markers determined by WB, were used to assess the quality of the purification. Lipid and protein analyses were performed using equal amounts of each fraction.

### 2.9. Analysis of the Endogenous Lipid Patterns

Total lipids were extracted and partitioned as previously described in order to obtain an aqueous phase mainly containing hydrophilic glycosphingolipids (gangliosides and salylated globosides) and an organic phase mainly containing neutral glycolipids, phospholipids, and apolar lipids [24,25,26]. Briefly, total lipids were extracted from lyophilized cell lysates with chloroform/methanol/water, 20:10:1 (*v*/*v*/*v*), and separated from the pellet by centrifugation at 13,000× *g* for 15 min, followed by a second extraction with chloroform/methanol, 2:1 (*v*/*v*). Total lipid extracts were subjected to a two-phase partitioning by adding 20% water, resulting in the separation of an aqueous phase and an organic phase. Samples obtained from the DRM preparation were dialyzed before lyophilization in order to eliminate sucrose. To correctly visualize neutral glycolipids [i.e., glucosylceramide (GlcCer), LacCer], phospholipids present in the organic phase were removed by a mild alkaline treatment. Lipids were separated by monodimensional HPTLC using different solvent systems: chloroform/methanol/0.2 aqueous CaCl_2_ (50:42:11, *v*/*v*/*v*) for hydrophilic glycosphingolipid analysis of the aqueous phase; chloroform/methanol/water (110:40:6, *v*/*v*/*v*) for neutral glycolipid analysis of the organic phase; hexane/ethylacetate (3:2, *v*/*v*) for cholesterol analysis of the organic phase; chloroform/methanol/acetic acid/water (30:20:2:2, *v*/*v*/*v*/*v*) for phospholipid and sphyngomyelin analysis of the organic phase; and hexane/chloroform/acetone/acetic acid (20:70:20:4, *v*/*v*/*v*/*v*) for ceramide analysis of the organic phase. After lipid separation, HPTLC was revealed using different spraying reagents: the anisaldehyde reagent was used to reveal neutral lipids, ceramide, and cholesterol; the Ehrlich reagent was used to visualize sialylated glycosphingolipids; and the phosphorus reagent was used to detect phospholipids and SM. Lipids were identified and quantified by co-migration with dosed lipid standards, such as ceramide, GlcCer, LacCer, globotriaosylceramide, phosphatidylcholine (PC), phosphatidylethanolamine (PE), phosphatidylinositol (PE), phosphatidylserine (PS), sphingomyelin, cholesterol, and the gangliosides GM3 and GM2, which were available in the laboratory. HPTLC was scanned and the band intensity quantified using the ImageJ software (2.14.0; Java 1.8.0_322, NIH, Bethesda, MD, USA; http://rsbweb.nih.gov/ij/ accessed on 1 July 2023) [22]; each band was normalized to the total protein content seeded in each lane. The concentration of each species was expressed as nmoles/mg proteins (see Table 1). The number of sialyl residues was taken into account for analysis of sialylated glycosphingolipid contents. In the graphs, data are presented as a percentage with respect to WT values, where the mean value of the WT was set to 1.0.

#### Sialidase Treatment

Aliquots of polar sphingolipids contained in the aqueous phase were incubated for 48 h at 37 °C with 0.02 units of *Vibrio cholerae* sialidase; then, the samples were dried and analyzed by HPTLC. To identify sialylated glycosphingolipids, HPTLC results acquired with Ehrlich and anisaldehyde reagents were compared, exploiting lipid standards that had been previously analyzed by mass spectrometry [24] (see Supplementary Figure S1).

### 2.10. Analysis of the Radioactive Lipid Patterns

Following DRM preparation, sucrose fractions and post-nuclear supernatants were dialyzed, lyophilized, and subjected to lipid extraction and partitioning, as described above. The radioactivity associated with the total lipids was determined by liquid scintillation counting by a beta-counter (PerkinElmer). Lipids were resolved by monodimen-sional HPTLC using chloroform/methanol/water (110:40:6, *v*/*v*/*v*) and identified by co-migration with standards available in the laboratory. Radioactive lipids were detected by radioactivity imaging (Beta-Imager TRacer Betaimager; BioSpace Laboratory, Nesles-la-Vallée, France) and quantified using M3 Vision software (1.0.8.1187; Bio-space Lab Inc.; https://biospacelab.com/ accessed on 1 July 2019); each band was normalized to the total radioactivity content seeded in each lane.

### 2.11. Evaluation of Enzymatic Activities in Cell Lysates

The enzymatic activities associated with total cell lysates were evaluated using an already described method [24,26,27,28]. The following MUB-derived fluorogenic substrates were employed: MUB-β-Glc for total beta-glucosidase (β-Glc), MUB-β-Gal for beta-galactosidase (β-Gal), MUB-α-Gal for alpha-galactosidase (α-Gal), MUG for total beta-hexosaminidase (β-Hex), MUGS for β-Hex A, and MUB-β-Man for beta-mannosidase (β-Man) (all obtained from Glycosynth, Warrington, UK). To assess sphingomyelinase (SMase) activity, HMUB-PC was used (Moscerdam Substrates, Oegstgeest, The Netherlands).

To evaluate beta-glucocerebrosidase (GCase) and non-lysosomal glucocerebrosidase (NLGase) activities, 20 μg quantities of cell lysates were pre-incubated in 96-well plates for 30 min at 23 °C with a reaction mixture containing 25 μL/well of McIlvaine buffer (0.4 M citric acid and 0.8 M Na_2_HPO_4_ (pH 6)); 5 nM AMP-Deoxynojirimycin, the specific inhibitor of NLGase, or 1 nM Conduritol B epoxide; the specific inhibitor of GCase; and water to a final volume of 75 μL/well. At the end of the pre-incubation, the reaction was started by adding 25 μL/well of 6 mM MUB-β-Glc.

To assess β-Gal, β-Hex, and β-Man activity, aliquots of cell lysates (10 μg for β-Gal and β-Hex and 20 μg for β-Man) were mixed with 25 μL/well of McIlvaine buffer (pH 5.2) containing the fluorogenic substrates at a final concentration of 500 μM; for SMase activity, 20 μg of cell lysate and 250 μM HMUB-PC were used. The total volume of 100 μL was then achieved by adding water, and the reaction mixtures were incubated at 37 °C under gentle shaking.

At different time-points, 10 μL of reaction mixture was transferred into a black microplate and mixed with 190 μL of 0.25 M glycine (pH 10.7) to stop the reaction. Then, 0.3% TX-100 was added to 0.25 M glycine (pH 10.7) for the SMase evaluation. The fluorescence (ex/em, 365/460 nm) was detected using a Victor microplate reader (PerkinElmer). Hexadecanoylamino-MUB and standard free MUB were utilized to measure substrate hydrolysis and create calibration curves. Data were expressed as nanomoles of converted substrate/h, normalized to mg of cell proteins with respect to WT values, where the mean value of the WT was set to 1.0.

### 2.12. Evaluation of Enzymatic Activity on the Surface of Living Cells

Cells were plated in 96-well microplates, cultured for 72 h, and the PM-associated activities of glycohydrolases were measured by a previously developed high-throughput live-cell-based assay [27]. To differentiate GCase from NLGase activity, cells were pre-incubated for 30 min at 23 °C in phenol red-free DMEM-F12 containing either 50 nM AMP-Deoxynojirimycin or 1 mM Conduritol B epoxide, respectively [26]. Artificial substrates were dissolved in phenol red-free DMEM-F12 at pH 6 to the following final concentrations: 6 mM MUB-β-Glc, 250 μM MUB-β-Gal, 500 μM MUB-α-Gal, 1 mM MUG, 1 mM MUGS, 1 mM MUB-β-Man, and 500 μM HMUB-PC. DMEM-containing specific substrate was added to living cells, which were incubated for 4 h at 37 °C. After incubation, 10 μL of each supernatant was transferred to a black 96-well microplate (Perkin Elmer), followed by the addition of 190 μL of 0.25 M glycine buffer at pH 10.7. Fluorescence was measured using a Victor microplate reader (Perkin Elmer) with excitation/emission wavelengths of 365/460 nm. Since the substrates cannot cross the PM, the detected fluorescence reflects activity exclusively of PM glycohydrolases. Enzymatic activity was quantified as nmoles of product/h normalized to mg of cell proteins and expressed with respect to WT (mean value of the WT set to 1.0).

### 2.13. Immunofluoresce Analysis

HEK293-T cells were cultured in 24 well-plates on glass coverslips coated with poly-L-lysine. Forty-eight hours later, the cells were washed with 1X PBS and fixed in 4% (*w*/*v*) paraformaldehyde dissolved in 1X PBS for twenty minutes at 23 °C. After permeabilization in blocking solution (5% horse serum in 1X PBS containing 0.2% *v*/*v* TX-100) for 1 h, the primary antibody, anti-Lamp-1 (1:10 in blocking buffer), was incubated for 2 h at 23 °C, and, finally, the secondary anti-mouse 488 Alexa secondary antibody (1:1000 in blocking buffer) was added for 1 h at 23 °C. Nuclei were counterstained using 0.0002% (*v*/*v*) Hoechst in 1X PBS (5 min, at 23 °C), and slides were mounted with Fluoromount-G reagent™ (Thermo Fisher Scientific, Waltham, MA, USA). Images were acquired using a NikonEclipse Ni upright microscope (100× magnification) equipped with a Nikon DIG-ITAL SIGHT DS-U1 CCD camera. For quantitative comparisons between WT and *ST3GAL5* KO cells, the same exposure periods were used to photograph each coverslip. Image analyses were performed using Fiji software (2.14.0; Java 1.8.0_322, NIH, Bethesda, MD, USA; http://rsbweb.nih.gov/ij/ accessed on 1 November 2023) [22]. Data were expressed as integrated density (mean fluorescence x area measured of each cell, IntDen; corrected for the background) over WT values (mean value of the WT set to 1.0) [29].

### 2.14. ATP Conent Analysis

Total ATP generation was assessed using the ATPlite kit, a luminescence ATP detection assay system (Perkin Elmer). Cells were plated in 96-well microplates, cultured for 48 h, and then lysed according to the manufacturer’s instructions. Luminescence was detected using a Victor microplate reader (PerkinElmer). ATP production was quantified by performing a calibration curve with ATP standards at known concentrations and normalized on total protein contents, as we previously reported [30]. Data were expressed as a percentage of the WT (mean value of the WT set to 1.0).

### 2.15. Statistical Analysis

Data were expressed as means ± SEMs and were analyzed for significance by the non-parametric Mann–Whitney test to compare two independent groups without assuming a normal distribution or one-way ANOVA when more than two groups were compared, as indicated in the figure legends. The analysis was performed with Prism software 10.3.0 (GraphPad Software, Inc., La Jolla, CA, USA; https://www.graphpad.com/, accessed on 1 July 2024). A *p* value < 0.05 was considered as significant.

## 3. Results

### 3.1. GM3SD Induces Changes in Lipid Composition

Given the role of GM3 as a precursor in ganglioside synthesis (i.e., a- and b-series), we aimed to investigate the impact of GM3SD on lipid composition in WT and *ST3GAL5* KO HEK293-T cells.

First, we performed a lipidomic analysis through HPTLC. Table 1 shows the lipidomic profile values of WT and *ST3GAL5* KO HEK293-T cells.

**Figure 1:**
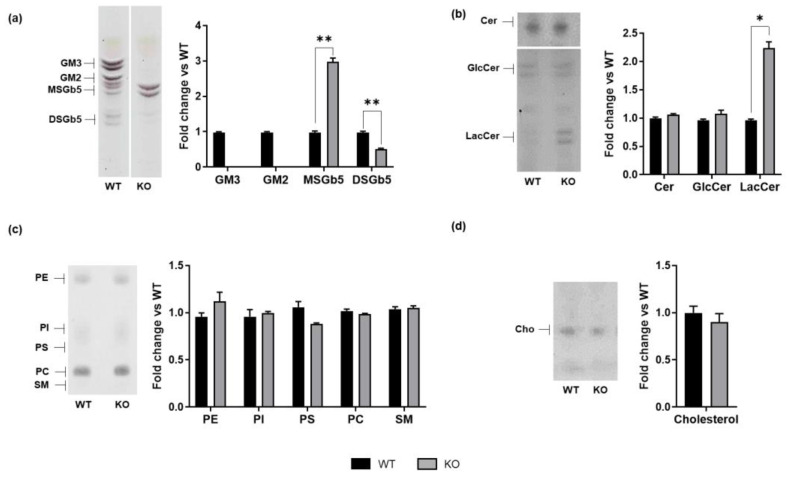
Lipid composition in WT and *ST3GAL5* KO cells. An endogenous lipid pattern was obtained by extraction with chloroform/methanol/water, 20:10:1 (*v*/*v*/*v*), and a two-phase partitioning of total cell lysates from WT and *ST3GAL5* KO cells. (**a**) The levels of sialylated glycosphingolipids were inferred by treating samples with *Vibrio cholerae* sialidase and visualized by Ehrlich reagent (for details, see Supplementary Figure S1), (**b**) neutral sphingolipids and ceramide were visualized by anisaldehyde reagent, (**c**) phospholipids and sphingomyelin were visualized by phosphorus reagent, and (**d**) cholesterol was visualized by anisaldehyde reagent. Lipids were identified by co-migration with specific lipid standards. The HPTLC images shown are representative of five independent experiments (*n* = 5) and have been equally adjusted for brightness and contrast. Data are displayed as fold changes with respect to WT cells (set to 1.0) and are presented as the mean ± SEM (* *p* < 0.05, ** *p* < 0.01 by Mann–Whitney test, WT vs. KO). SM: sphingomyelin; Cer: ceramide; Cho: cholesterol.

Specifically, WT cells expressed the gangliosides GM2 and GM3, as well as the globosides MSGb5 and DSGb5. In contrast, as expected, *ST3GAL5* KO cells showed a complete absence of a-series gangliosides (Figure 1a). Importantly, the analysis revealed an approximately 1.5-fold increase in LacCer levels, the substrate of ST3GAL5, along with increased levels of MSGb5 globoside and reduced levels of DSGb5 globoside in *ST3GAL5* KO cells compared to WT cells (Figure 1a,b). These findings partially align with previous studies on plasma, fibroblasts, and neural crest cells derived from induced pluripotent stem cells of patients carrying loss-of-function mutations in the *ST3GAL5* gene [12,15,16,31]. Meanwhile, sphingomyelin levels in *ST3GAL5* KO cells were comparable to those found in WT cells (Figure 1c). Similarly, no detectable alteration in total cholesterol was observed upon GM3S depletion (Figure 1d). These findings suggest that GM3S-deficient cells accumulate its substrate, LacCer, which is partially channeled towards the synthesis of MSGb5 globoside. The reduced DSGb5 content may instead result from its continuous conversion into MSGb5.

Since sphingolipids, together with cholesterol, are essential elements of lipid rafts [4], we decided to further investigate the potential effects of GM3SD on membrane lipid organization and composition.

**Figure 2:**
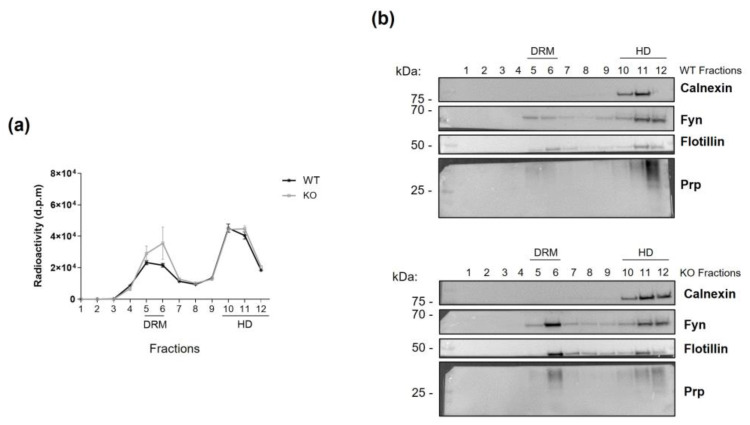
Purification of DRM fractions. Cell lysates obtained from WT and *ST3GAL5* KO HEK293-T cells fed with [1-^3^H] sphingosine to the steady state were subjected to ultracentrifugation in a sucrose gradient to isolate plasma membrane microdomains. Twelve fractions were collected, starting from the top of the tube, with fractions 5–6 corresponding to the DRM fractions and fractions 10–12 to the HD fractions. (**a**) The radioactivity distribution in the different fractions was evaluated by liquid scintillation counting by a beta-counter and expressed as disintegrations per minute (d.p.m.). (**b**) Levels of specific protein markers in the WT (top) and *ST3GAL5* KO (bottom) cells in the fractions were analyzed by WB to ensure proper DRM purification. Calnexin was used as an HD marker, while flotillin, Fyn proto-oncogene Src Family Tyrosine Kinase (Fyn), and prion protein (Prp) were used as DRM markers. Data are representative of five independent experiments (*n* = 5).

To this end, we isolated lipid raft-enriched portions of cell membranes as DRM fractions. Cells were metabolically labeled at the steady state using the radioactive precursor [1-^3^H]sphingosine, and after 24 h, DRM fractions were isolated from both WT and *ST3GAL5* KO cells. DRM purification was validated by assessing the radioactivity distribution and verifying the correct localization of specific protein markers in the different fractions, as shown in Figure 2.

**Figure 3:**
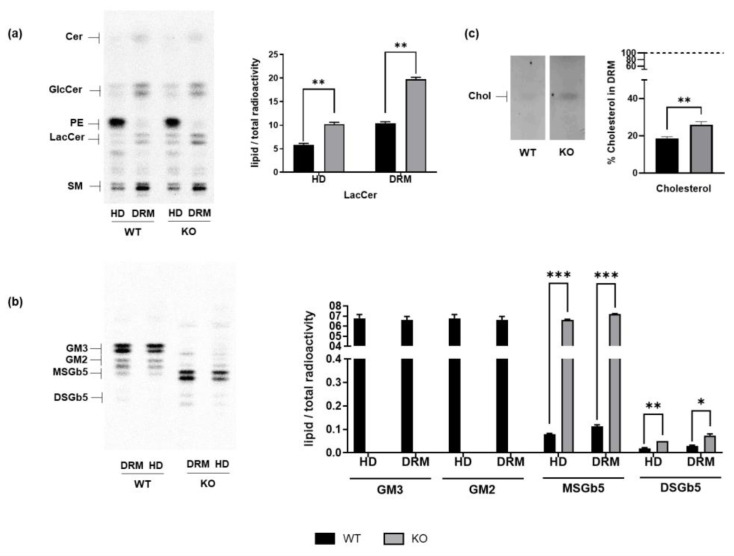
DRM lipid composition in WT and *ST3GAL5* KO cells. Cell sphingolipids were metabolically labelled by [1-^3^H]sphingosine. Following cell lysis with 1% TX-100 at 4 °C, the HD and lipid rafts (DRMs) were isolated by ultracentrifugation on a discontinuous sucrose gradient. Extracted lipids, obtained from the organic phase and aqueous phase, corresponding to equal radioactivity amounts, were separated using HPTLC, visualized using digital autoradiography, and measured by M3 software (1.0.8.1187; Biospace Lab Inc; https://biospacelab.com/, accessed on 1 July 2019). (**a**) LacCer and (**b**) ganglioside (GM3 and GM2) and globoside (MSGb5 and DSGb5) levels were obtained by quantifying the specific lipid bands and normalizing them over the total radioactivity of the lipid species in the lane. (**c**) Cholesterol levels were visualized by means of anisaldehyde reagent and expressed as percentages of total cholesterol (HD + DRM). The HPTLC images shown are representative of five independent experiments (*n* = 5) and have been equally adjusted for brightness and contrast. Values are expressed as means ± SEMs (*** *p* < 0.001, ** *p* < 0.01, * *p* < 0.05 by Mann–Whitney test, WT vs. KO). Cho: cholesterol.

As shown in Figure 3, LacCer, MSGb5, and DSGb5 levels were significantly elevated both in HD (containing non-raft-associated proteins and lipids) and DRM fractions in *ST3GAL5* KO cells with respect to WT cells. The increased presence of globosides in lipid rafts may act as a compensatory mechanism to balance the loss of negative charges typically provided by sialylated glycosphingolipids at the membrane.

Importantly, *ST3GAL5* KO cells exhibited a substantial increase in cholesterol in DRMs (Figure 3c). This would determine a rise in lipid rafts’ rigidity, affecting the ability of cells to communicate through signaling molecules and leading to changes in cell function.

### 3.2. GM3SD Determines Alterations in Glycosphingolipid Glycohydrolase Activity

Based on the observed alterations in the glycosphingolipid pattern, we decided to evaluate the activity of enzymes involved in their catabolism. This breakdown occurs on the luminal side of late endosomes and lysosomes, where it is stepwise orchestrated by acidic hydrolases, with the assistance of small glycoproteins that bind to lipids [5,6,9].

Starting from the cell lysate, thus focusing primarily on the lysosomal compartment, we assessed the activity of the following enzymes: α-Gal, generating LacCer from Gb3 globoside; β-Gal, producing GlcCer from LacCer; β-Hex, generating Gb3 from Gb4 globoside; GCase and total β-Glc, producing ceramide from GlcCer; and SMase, catalyzing the breakdown of sphingomyelin to ceramide and phosphocholine.

**Figure 4:**
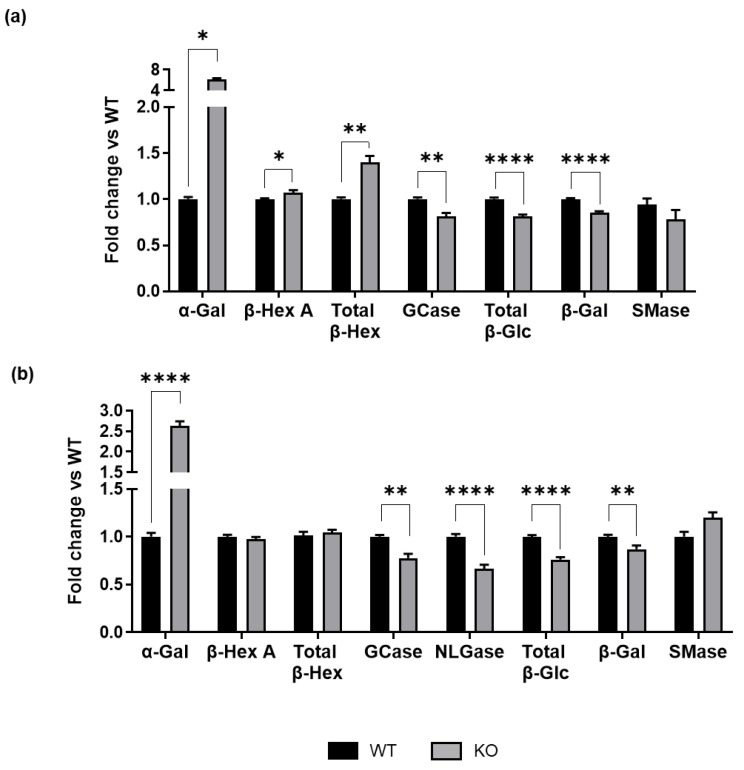
Activity of sphingolipid hydrolases in WT and *ST3GAL5* KO cells. Enzymatic activity of (**a**) lysosomal and (**b**) PM-associated hydrolases. Data are displayed as fold changes with respect to WT cells (set to 1.0) and are presented as the means ± SEMs of six independent experiments (**** *p* < 0.0001, ** *p* < 0.01, * *p* < 0.05 by Student’s *t*-test, WT vs. KO, *n* = 6).

As shown in Figure 4a, we observed altered activity in several of the tested enzymes. In *ST3GAL5* KO cells compared to WT cells, α-Gal and β-Hex were found to be hyperactivated, with α-Gal displaying an over 6-fold increase in activity. On the contrary, GCase and total β-Glc, as well as β-Gal, were hypoactivated. Specifically, among glucosidases, GCase activity was roughly 30% lower in *ST3GAL5* KO cells than in WT cells. No alteration was detected in SMase activity. These findings demonstrate that defects in GM3 ganglioside synthesis impact the activity of enzymes involved in glycosphingolipid catabolism. Among them, the unexpected increase in α-Gal activity may further contribute to LacCer accumulation.

Given that alterations in lipid contents were also observed in the PM (Figure 3), we measured the activity of glycohydrolases located at the cell surface of living cells. As is now well established, these catabolic enzymes translocate through a process of fusion between lysosomes and the PM, allowing them to extend their activity beyond the confines of lysosomes [26,27,32]. Except for β-Hex, whose activity was similar between the two experimental groups, we found altered enzymatic activities, consistent with those measured in whole-cell lysates (Figure 4b). Specifically, α-Gal activity was roughly 3-fold higher in *ST3GAL5* KO cells, while β-Gal and glucosidase activities were reduced compared to WT cells. In addition, a decrease in the activity of NLGase was found on the cell surface, where it primarily operates [26]. The deregulation of hydrolases on the PM corresponds well with the altered lipid profile, particularly with the significant increase in LacCer levels in lipid raft domains, which are highly enriched in the PM (Figure 3). These results indicate that deregulation of the ganglioside biosynthetic pathway, due to the absence of GM3S activity, leads to changed activity of enzymes responsible for glycosphingolipid catabolism at the PM as well.

### 3.3. GM3SD Prompts a Secondary Alteration in the Lysosomal Compartment

**Figure 5:**
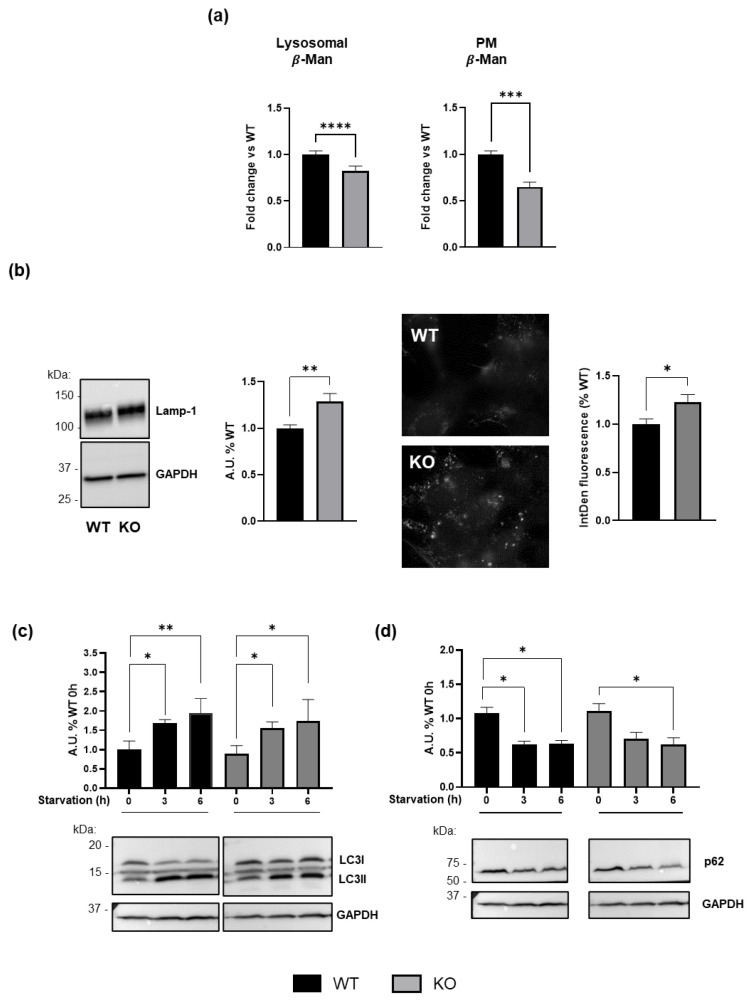
Consequences of GM3SD for lysosomal homeostasis. (**a**) Enzymatic activity of lysosomal and PM-associated β-Man hydrolase in WT and *ST3GAL5* KO cells. (**b**) Lamp-1 protein levels were evaluated by Western blot (WB) analysis (left) and immunofluorescence (right, 100× magnification) in WT and *ST3GAL5* KO cells. WB analysis of (**c**) LC3 and (**d**) p62 proteins in cells subjected to amino acid starvation in WT and *ST3GAL5* KO cells. WB data were obtained by normalizing the signal intensity of LC3-II and p62 to that of GAPDH, used as a loading control; immunofluorescence results are expressed as the integrated density (IntDen, mean fluorescence x area measured of each cell; corrected for the background). Images shown are representative of five independent experiments and have been equally adjusted for brightness and contrast. Histograms depict the means ± SEMs of values obtained from five independent experiments (*n* = 5) and expressed as percentages with respect to WT controls (set to 1.0; A.U.: arbitrary units). Data were compared by Mann–Whitney tests ((**a**,**b**) **** *p* < 0.0001, *** *p* < 0.001, ** *p* < 0.01, * *p* < 0.05, WT vs. KO) and one-way ANOVA followed by Tukey’s post hoc test ((**c**,**d**) ** *p* < 0.01, * *p* < 0.05 vs. WT before starvation, 0 h).

To determine whether GM3SD could induce secondary alterations in the lysosomal compartment not directly related to glycolipid metabolism, we evaluated the activity of β-Man in WT and *ST3GAL5* KO cells. This enzyme catalyzes the hydrolysis of terminal, non-reducing β-D-mannose residues in β-D-mannosides linked to glycoproteins within the lysosomes. Our analysis revealed reduced activation of β-Man in lysosomes of *ST3GAL5* KO cells (Figure 5a). Notably, the same downregulation was detected also at the PM level (Figure 5a). This observation suggests a potential deregulation in lysosomal homeostasis and an alteration in the lysosome–PM axis.

We further assessed lysosome abundance and identified an increase in lysosomal mass through immunoblotting and immunofluorescence staining for Lamp-1 (Figure 5b).

To evaluate lysosomal function in greater detail, we examined the autophagic machinery by subjecting cells to starvation. As shown in Figure 5c, levels of microtubule-associated proteins 1A/1B light chain 3B and LC3-II increased significantly upon starvation in both WT and *ST3GAL5* KO cells, indicating autophagosome formation and potentially enhanced autophagic activity. Accordingly, we observed a reduced level of the autophagy substrate protein sequestosome 1, p62 (Figure 5d), indicating effective autophagosome degradation [33].

Thus, despite the observed increase in lysosomal biogenesis, we did not observe an impairment in the autophagy system. This is likely because the cellular model used here is capable of division, which may help mitigate cellular damage via mitosis.

### 3.4. GM3SD Induces Bioenergetic Deficiency

**Figure 6:**
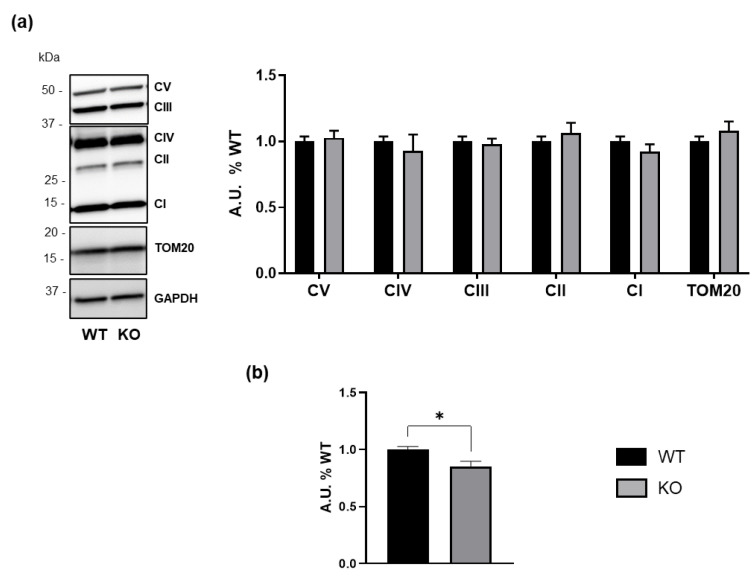
Effect of GM3SD on mitochondrial proteins and energy production. (**a**) The levels of mitochondrial proteins in WT and *ST3GAL5* KO cells evaluated by WB. The WB images shown are representative of six independent experiments. Histograms display the levels of Oxphos complexes and TOM20. WB data were obtained by dividing the signal intensity of the band associated with specific proteins by that of GAPDH, used as the loading control. (**b**) Total ATP levels of WT and *ST3GAL5* KO cells. Data are expressed as the mean ± SEM of the percentage of WT controls (set to 1.0) and were obtained from six independent experiments (* *p* < 0.05 by Mann–Whitney test, WT vs. KO, *n* = 6).

Considering the altered mitochondrial function observed in samples from patients with GM3SD [12], we decided to investigate whether this phenotype could be replicated in our experimental model. Consistent with data obtained from analyses on murine *St3gal5*-null brains, mitochondrial protein levels were unaffected by GM3SD, suggesting no change in mitochondrial mass (Figure 6a).

However, we observed reduced ATP levels in *ST3GAL5* KO cells compared to WT controls (Figure 6b), suggesting an energetic imbalance.

## 4. Discussion

Gangliosides play a crucial role in maintaining nervous system homeostasis, with both their accumulation and depletion being detrimental [1,2,3,4,6,8,9]. Disruption to ganglioside metabolism is linked to various neurological disorders, ranging from neurodevelopmental to neurodegenerative conditions [2,6,10].

GM3 ganglioside serves as the precursor of complex brain gangliosides, such as GM1a, GD1a, GD1b, and GT1b [1,2,5,6]. Loss-of-function mutations in the *ST3GAL5* gene, the gene encoding GM3 synthase, lead to a complete absence of gangliosides, causing a severe infantile-onset epileptic syndrome in humans [11,12,13,14,15,16,17,31].

Unlike humans [16,18], *St3gal5*-null mice do not fully recapitulate the severe symptoms observed in patients. This discrepancy may be due to species-specific differences in variations in B4GALNT1 activity, which allow mice to compensate for the absence of a- and b-series gangliosides by producing 0-series gangliosides (i.e., GM1b and GD1α) from LacCer [2,34]. However, in human-derived cells, LacCer accumulation is observed, suggesting that its conversion to 0-series gangliosides is inefficient [12,15,16,31,34,35]. Additionally, higher globoside levels are detected in human samples with *ST3GAL5* mutations, potentially contributing to disease pathogenesis through inhibition of GM3 synthase via AUTS2 repression [36]. Indeed, AUTS2 works by activating GM3 synthase, promoting ganglioside synthesis and regulating glycosphingolipid reprogramming, linking it to neurodevelopment and neuropathology.

**Figure 7:**
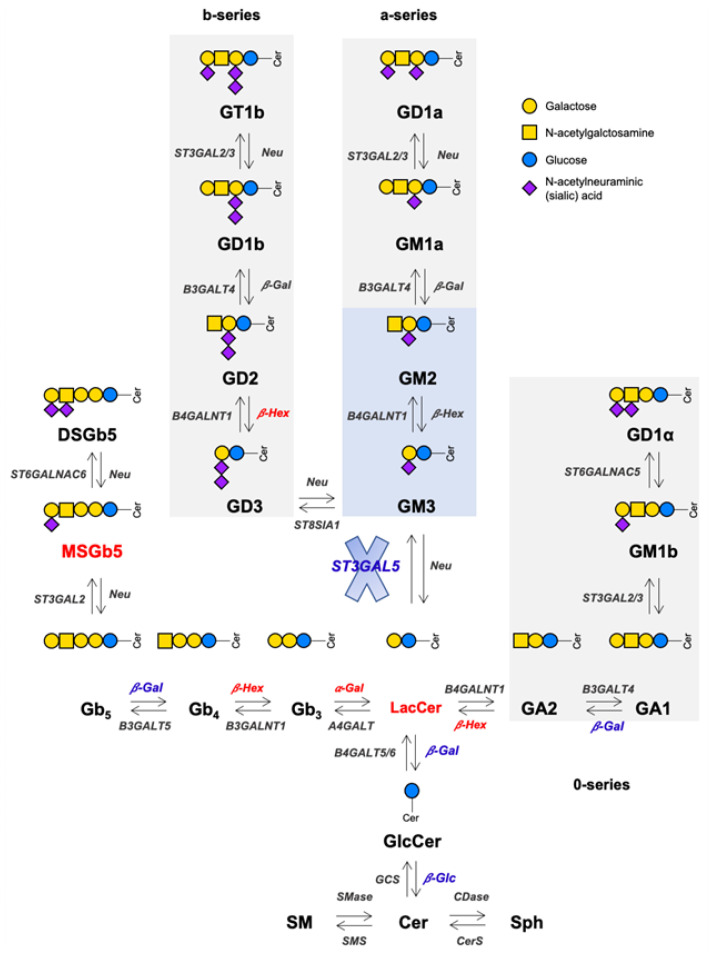
Effect of GM3SD on sphingoid-base lipid metabolism in HEK293-T cells. GM3S metabolizes LacCer into GM3, the precursor for subsequent a- and b-series gangliosides. Alternatively, LacCer can be used as a substrate for the synthesis of 0-series gangliosides. Lipid species overexpressed in *ST3GAL5* KO cells and catabolic hyperactivated enzymes are shown in red, while the downregulated ones are shown in blue. Enzymes that were not tested in this study are indicated in grey. The light-blue box highlights the a-series gangliosides that are not expressed in *ST3GAL5* KO cells; the grey box indicates b-series and 0-series gangliosides that are not expressed in WT or *ST3GAL5* KO cells. Oligosaccharide sugar symbols were selected according to Varki et al. [43]. ST3GAL2: ST3 beta-galactoside alpha-2,3-sialyltransferase 2; ST3GAL3: ST3 beta-galactoside alpha-2,3-sialyltransferase 3; ST3GAL5: ST3 beta-galactoside alpha-2,3-sialyltransferase 5; Neu: neuraminidase; B3GALT4: beta-1,3-galactosyltransferase 4; B4GALNT1: be-ta-1,4-N-acetyl-galactosaminyltransferase 1; ST8SIA1: ST8 alpha-N-acetyl-neuraminide alpha-2,8-sialyltransferase 1; ST6GALNAC5: ST6 N-acetylgalactosaminide alpha-2,6-sialyltransferase 5; ST6GALNAC6: ST6 N-acetylgalactosaminide alpha-2,6-sialyltransferase 6; B3GALT5: beta-1,3-galactosyltransferase 5; B3GALNT1: be-ta-1,3-N-acetylgalactosaminyltransferase 1; A4GALT: alpha 1,4-galactosyltransferase; GCS: glucosylceramide synthase; CDase: ceramidase; CerS: ceramide synthase; SMS: sphingomyelin synthase; Cer: ceramide; SM: sphingomyelin.

Our findings align with patient-derived data, demonstrating a significant increase in LacCer and MSGb5 globoside levels in *ST3GAL5* KO HEK293-T cells (Figure 1 and Figure 3). Notably, lipid rafts showed increased MSGb5 and DSGb5, which may have compensated for the loss of negatively charged sialylated glycosphingolipids, stabilizing membrane interactions. Furthermore, 0-series and b-series gangliosides were undetectable, confirming that these lipid species were not produced in the HEK293-T cells (Figure 7).

Glycosphingolipid synthesis in the Golgi apparatus is traditionally regulated by glycosyltransferases and transporter proteins, such as ceramide-transfer protein (CERT) and the lipid-transfer protein FAPP2 [1,5,6,7,8]. However, their intracellular trafficking also modulates PM composition, independently of enzyme levels. Coordinated activity between biosynthetic and lysosomal enzymes is essential for maintaining glycosphingolipid homeostasis [6,9,27].

In GM3S-lacking cells, we observed altered lysosomal enzyme activities (Figure 4), which likely exacerbated LacCer accumulation (Figure 7). Specifically, α-Gal hyperactivation (Figure 4) contributes to excessive LacCer production from Gb3 globoside (Figure 7), potentially triggering oxidative stress and inflammation [37].

Glycosphingolipid composition is also influenced by PM-associated enzymatic activity, including both catabolic enzymes (i.e., sialidases, β-Hex, β-Gal, and β-Glc) and biosynthetic enzymes (i.e., sialyltransferases) [26,27,38,39]. Accordingly, our analysis of PM catabolic enzyme activities (Figure 4) showed a deregulation in *ST3GAL5* KO cells, except for β-Hex, compared to WT cells, mirroring the alterations in the lysosomal compartment. Our findings revel that α-Gal activity was also significantly elevated at the PM, further increasing LacCer levels within lipid rafts (Figure 4). This, combined with higher cholesterol levels (Figure 3), suggests that lipid rafts become more ordered and rigid, potentially impacting membrane signaling pathways [40].

The reduced β-Man activity in both lysosomes and PM compartments (Figure 4) suggests a connection between lysosomal dysfunction and PM remodeling, likely dependent on lysosome–PM fusion. The PM–lysosomal axis acts as a pathogenetic mechanism that disrupts PM structure and function by incorporating lysosomal enzymes and lipids into the PM [26,41]. This alters lipid rafts, key regulators of intracellular signaling, and promotes the release of lysosomal contents, potentially triggering inflammatory responses and contributing to neurodegenerative disease progression.

Of interest, the reduced β-Man activity has already been reported for mannosidosis, a lysosomal storage disease affecting the nervous system [42].

Increased Lamp-1 levels (Figure 5) in *ST3GAL5* KO cells suggest an induction of lysosomal biogenesis as a cellular response to lysosomal stress [44]. While autophagy appeared to be functional in GM3S-deficient cells (Figure 5), autophagy impairment in neurons cannot be ruled out, as post-mitotic cells rely on autophagy for damage control. Notably, elevated LacCer levels correlate with increased levels of the defective-autophagy marker p62 [45].

Gangliosides are known to modulate mitochondrial homeostasis, suggesting a potential link between GM3S deficiency and secondary mitochondrial dysfunction. In fibroblast and liver samples from *ST3GAL5*-mutant patients, respiratory chain dysfunction, reduced mitochondrial membrane potential in basal and non-phosphorylating conditions, and increased apoptosis upon rotenone exposure were observed [12]. Additionally, altered metabolic activity due to elevated glucose utilization has been reported in mice lacking GM3S [46].

Similarly, our findings indicate that HEK293-T *ST3GAL5* KO cells exhibit impaired ATP production (Figure 6), despite unaltered Oxphos protein levels (Figure 6). This suggests a bioenergetic alteration potentially linked to mitochondrial dysfunction, in line with reports that LacCer accumulation promotes ROS production and impairs mitochondrial respiration in other disease models [47].

## 5. Conclusions and Future Perspectives

Our findings confirm that GM3SD disrupts lipid homeostasis, increasing LacCer and sialylated Gb5 levels, while altered lysosomal and PM enzyme activities contribute to these abnormalities. Additionally, lipid raft remodeling and bioenergetic dysfunction suggest broader cellular consequences of *STGAL5* absence. Accordingly, given the dominant-negative effects in some *STGAL5* mutations [48], it is plausible that the absence of GM3 synthase may impact other cellular mechanisms, warranting further investigation into its potential non-canonical functions.

We acknowledge that HEK293-T cells are not neuronal models, which limits their ability to fully recapitulate GM3SD, a severe neurodevelopmental disorder. However, these cells express neuronal markers, including neurofilaments, and have been used to study genetic mutations linked to neurological disease [19].

Importantly, our *STGAL5* KO model replicates key findings from patient-derived cells, making it a valuable pre-screening platform for identifying molecular mechanisms of GM3SD and testing potential therapeutic agents. Recent studies have shown that oral GM3 and GD3 supplementation supports growth and cognitive development in a GM3SD mouse model [49]. However, the mechanism behind these improvements remains unclear, as direct brain incorporation of exogenous gangliosides has not been confirmed. Further studies using *ST3GAL5* KO HEK293-T cells could provide insights into the molecular pathways involved in these therapeutic effects.

Overall, our study highlights the multifaceted impact of GM3S deficiency, revealing lipid, lysosomal, and bioenergetic alterations in HEK293-T *ST3GAL5* KO cells. These findings provide new insights into GM3SD pathogenesis and establish this KO model as a useful tool for further research and potential therapeutic screening.

## Figures and Tables

**Table 1:** Lipid contents in WT and *ST3GAL5* KO cells.

Lipids	WT	*ST3GAL5* KO
GM3	0.72 ± 0.03	ND
GM2	0.50 ± 0.01	ND
MSGb5	0.29 ± 0.01	0.85 ± 0.01 **
DSGb5	0.12 ± 0.01	0.06 ± 0.01 **
Cholesterol	29.53 ± 3.79	26.88 ± 4.18
Ceramide	1.89 ± 0.06	1.99 ± 0.09
LacCer	0.64 ± 0.13	1.43 ± 0.29 *
GlcCer	0.93 ± 0.11	0.99 ± 0.11
PE	21.47 ± 2.90	23.63 ± 2.68
PI	10.80 ± 1.48	10.75 ± 1.39
PS	11.02 ± 1.23	9.70 ± 1.02
PC	40.50 ± 3.10	39.98 ± 2.95
Sphingomyelin	5.31 ± 0.95	5.55 ± 0.99

Ganglioside (GM3 and GM2), globoside (MSGb5 and disialyl-globopentaosylceramide (DSGb5)), ceramide, cholesterol, and glycerophospholipid (including PE, PS, PC, and PI) and sphingomyelin contents were evaluated by HPTLC in WT and *ST3GAL5* KO cells. Data obtained from five independent experiments (*n* = 5) are expressed as the mean ± standard error of the mean (SEM) nmol/mg cell protein (* *p* < 0.05, ** *p* < 0.01 by Mann–Whitney test, WT vs. KO). ND: not detectable.

## Data Availability

The original contributions presented in the study are included in the article and Supplementary Materials, further inquiries can be directed to the corresponding authors.

## Supplementary Materials

The following supporting information can be downloaded at https://www.mdpi.com/article/10.3390/biomedicines13040843/s1, Figure S1: Identification of sialylated lipid patterns in WT and *ST3GAL5* KO HEK293-T cells.

## Abbreviations

The following abbreviations are used in this manuscript:

The glycosphingolipid nomenclature is in accordance with IUPAC-IUBB recommendations [50].	
α-Gal	Alpha-galactosidase
ATP	Adenosine triphosphate
A.U.	Arbitrary unit
β-Gal	Beta-galactosidase
β-Glc	Beta-glucosidase
β-Hex	Beta-hexosaminidase
β-Man	Beta-mannosidase
BSA	Bovine serum albumin
DMEM	Dulbecco’s modified eagles’ medium
d.p.m.	Disintegrations per minute
DSGb5	Neu5Acα3Galβ3(NeuAcα6)GalNacβ3Galα4Galβ1GlcCer, disialyl-Gb5 globoside, disialyl-globopentaosylceramide
DRM	Detergent-resistant membrane
EDTA	Ethylenediaminetetraacetic acid
GAPDH	Glyceraldehyde-3-phosphate dehydrogenase
GA2	GalNacβ4Galβ4GlcCer
GA1	Galβ3GalNacβ4Galβ4GlcCer
Gb3	Galα4Galβ1GlcCer, Gb3 globoside, globotriaosylceramide
Gb4	GalNacβ3Galα4Galβ1GlcCer, Gb4 globoside, globotetraosylceramide
Gb5	Galβ3GalNacβ3Galα4Galβ1GlcCer, Gb5 globoside
Gb3	Galα4Galβ1GlcCer, Gb3 globoside
GCase	Beta-glucocerebrosidase
GD1a	Neu5Acα3Galβ3GalNAcβ4(Neu5Acα3)Galβ4GlcCer, GD1a ganglioside
GD1b	Galβ3GalNAcβ4(Neu5Acα8Neu5Acα3)Galβ4GlcCer, GD1b ganglioside
GD1α	Neu5Acα3Galβ3(Neu5Acα6)GalNAcβ4Galβ4GlcCer, GD1 alpha ganglioside
GD2	GalNAcβ4(Neu5Acα8Neu5Acα3)Galβ4GlcCer, GD2 ganglioside
GD3	Neu5Acα8(Neu5Acα3)Galβ4GlcCer, GD3 ganglioside
GlcCer	Glucosylceramide
GM1a	Galβ3GalNAcβ4(Neu5Acα3)Galβ4GlcCer, GM1a ganglioside
GM1b	Neu5Acα3Galβ3GalNAcβ4Galβ4GlcCer, GM1b ganglioside
GM2	GalNAcβ4(Neu5Acα3)Galβ4GlcCer, GM2 ganglioside
GM3	Neu5Acα3Galβ4GlcCer, GM3 ganglioside
GM3S	GM3 synthase
GM3SD	GM3 Synthase Deficiency
GT1b	Neu5Acα3Galβ3GalNAcβ4(Neu5Acα8Neu5Acα3)Galβ4GlcCer, GT1b ganglioside
HMUB-PC	6-hexadecanoylamino 4-MU-phosphoryl-choline
HD	High-density fractions
HEK293-T	Human embryonic kidney 293-T
HPTLC	High-performance thin-layer chromatography
IntDen	Integrated density
KO	Knockout
LC3	Microtubule-associated proteins 1A/1B light chain 3A
LacCer	Galβ4GlcCer, lactosylceramide
Lamp-1	Lysosomal associated membrane protein 1
LC3	Microtubule-associated proteins 1A/1B light chain 3A
MSGb5	Neu5Acα3Galβ3GalNacβ3Galα4Galβ1GlcCer, monosialyl-globoside Gb5, monosialyl-globopentaosylceramide
MUB-α-Gal	4-methylumbelliferyl α-D-galactopyranoside
MUB-β-Gal	4-methylumbelliferyl β-D-galactopyranoside
MUB-β-Glc	4-methylumbelliferyl β-D-glucopyranoside
MUB-β-Man	4-methylumbelliferyl-β-D-mannopyranoside
MUG	4-methylumbelliferyl N-acetyl-β-D-glucosaminide
MUGS	4-methylumbelliferyl N-acetyl-β-D-glucosaminide-6-sulfate
NaCl	Sodium chloride
Na_3_VO_4_	Sodium orthovanadate
NLGase	Non-lysosomal glucosylcerebrosidase
Oxphos	Oxidative phosphorylation
PBS	Phosphate-buffered saline
PC	Phosphatidylcholine
PE	Phosphatidylethanolamine
PI	Phosphatidylinositol
PM	Plasma membrane
PMSF	Phenylmethanesulfonyl fluoride
Prp	Prion protein
PS	Phosphatidylserine
PVDF	Polyvinylidene difluoride
p62	Ubiquitin-binding protein p62
RRID	Research resource identifier
SDS	Sodium dodecyl sulfate
SM	Sphingomyelin
SEM	Standard error of the mean
SMAse	Sphingomyelinase
TBS-T	Tris-buffered saline containing 0.1% tween-20
TOM20	Translocase of outer mitochondrial membrane 20
TX-100	Triton X-100
WB	Western blotting
WT	Wild type

## References

[1] Ledeen R., Wu G. (2018). Gangliosides of the Nervous System. Methods Mol. Biol..

[2] Sipione S., Monyror J., Galleguillos D., Steinberg N., Kadam V. (2020). Gangliosides in the Brain: Physiology, Pathophysiology and Therapeutic Applications. Front. Neurosci..

[3] Ohmi Y., Ohkawa Y., Yamauchi Y., Tajima O., Furukawa K., Furukawa K. (2012). Essential roles of gangliosides in the formation and maintenance of membrane microdomains in brain tissues. Neurochem. Res..

[4] Pike L.J. (2003). Lipid rafts: Bringing order to chaos. J. Lipid Res..

[5] D’Angelo G., Capasso S., Sticco L., Russo D. (2013). Glycosphingolipids: Synthesis and functions. FEBS J..

[6] Sandhoff R., Sandhoff K. (2018). Emerging concepts of ganglioside metabolism. FEBS Lett..

[7] Merrill A.H. (2011). Sphingolipid and glycosphingolipid metabolic pathways in the era of sphingolipidomics. Chem. Rev..

[8] Yu R.K., Nakatani Y., Yanagisawa M. (2009). The role of glycosphingolipid metabolism in the developing brain. J. Lipid Res..

[9] Quinville B.M., Deschenes N.M., Ryckman A.E., Walia J.S. (2021). A Comprehensive Review: Sphingolipid Metabolism and Implications of Disruption in Sphingolipid Homeostasis. Int. J. Mol. Sci..

[10] Li T.A., Schnaar R.L. (2018). Congenital Disorders of Ganglioside Biosynthesis. Prog. Mol. Biol. Transl. Sci..

[11] Simpson M.A., Cross H., Proukakis C., Priestman D.A., Neville D.C., Reinkensmeier G., Wang H., Wiznitzer M., Gurtz K., Verganelaki A. (2004). Infantile-onset symptomatic epilepsy syndrome caused by a homozygous loss-of-function mutation of GM3 synthase. Nat. Genet..

[12] Fragaki K., Ait-El-Mkadem S., Chaussenot A., Gire C., Mengual R., Bonesso L., Beneteau M., Ricci J.E., Desquiret-Dumas V., Procaccio V. (2013). Refractory epilepsy and mitochondrial dysfunction due to GM3 synthase deficiency. Eur. J. Hum. Genet..

[13] Lee J.S., Yoo Y., Lim B.C., Kim K.J., Song J., Choi M., Chae J.H. (2016). GM3 synthase deficiency due to ST3GAL5 variants in two Korean female siblings: Masquerading as Rett syndrome-like phenotype. Am. J. Med. Genet. A.

[14] Heide S., Jacquemont M.L., Cheillan D., Renouil M., Tallot M., Schwartz C.E., Miquel J., Bintner M., Rodriguez D., Darcel F. (2022). GM3 synthase deficiency in non-Amish patients. Genet. Med..

[15] Boccuto L., Aoki K., Flanagan-Steet H., Chen C.F., Fan X., Bartel F., Petukh M., Pittman A., Saul R., Chaubey A. (2014). A mutation in a ganglioside biosynthetic enzyme, ST3GAL5, results in salt & pepper syndrome, a neurocutaneous disorder with altered glycolipid and glycoprotein glycosylation. Hum. Mol. Genet..

[16] Bowser L.E., Young M., Wenger O.K., Ammous Z., Brigatti K.W., Carson V.J., Moser T., Deline J., Aoki K., Morlet T. (2019). Recessive GM3 synthase deficiency: Natural history, biochemistry, and therapeutic frontier. Mol. Genet. Metab..

[17] Wang H., Wang A., Wang D., Bright A., Sency V., Zhou A., Xin B. (2016). Early growth and development impairments in patients with ganglioside GM3 synthase deficiency. Clin. Genet..

[18] Inamori K., Inokuchi J. (2022). Ganglioside GM3 Synthase Deficiency in Mouse Models and Human Patients. Int. J. Mol. Sci..

[19] Shaw G., Morse S., Ararat M., Graham F.L. (2002). Preferential transformation of human neuronal cells by human adenoviruses and the origin of HEK 293 cells. FASEB J..

[20] Nihei W., Nagafuku M., Hayamizu H., Odagiri Y., Tamura Y., Kikuchi Y., Veillon L., Kanoh H., Inamori K.I., Arai K. (2018). NPC1L1-dependent intestinal cholesterol absorption requires ganglioside GM3 in membrane microdomains. J. Lipid Res..

[21] Schiumarini D., Loberto N., Mancini G., Bassi R., Giussani P., Chiricozzi E., Samarani M., Munari S., Tamanini A., Cabrini G. (2017). Evidence for the Involvement of Lipid Rafts and Plasma Membrane Sphingolipid Hydrolases in Pseudomonas aeruginosa Infection of Cystic Fibrosis Bronchial Epithelial Cells. Mediators Inflamm..

[22] Schneider C.A., Rasband W.S., Eliceiri K.W. (2012). NIH Image to ImageJ: 25 years of image analysis. Nat. Methods.

[23] Chiricozzi E., Ciampa M.G., Brasile G., Compostella F., Prinetti A., Nakayama H., Ekyalongo R.C., Iwabuchi K., Sonnino S., Mauri L. (2015). Direct interaction, instrumental for signaling processes, between LacCer and Lyn in the lipid rafts of neutrophil-like cells. J. Lipid Res..

[24] Valsecchi M., Cazzetta V., Oriolo F., Lan X., Piazza R., Saleem M.A., Singhal P.C., Mavilio D., Mikulak J., Aureli M. (2020). APOL1 polymorphism modulates sphingolipid profile of human podocytes. Glycoconj. J..

[25] Prinetti A., Prioni S., Chiricozzi E., Schuchman E.H., Chigorno V., Sonnino S. (2011). Secondary alterations of sphingolipid metabolism in lysosomal storage diseases. Neurochem. Res..

[26] Samarani M., Loberto N., Solda G., Straniero L., Asselta R., Duga S., Lunghi G., Zucca F.A., Mauri L., Ciampa M.G. (2018). A lysosome-plasma membrane-sphingolipid axis linking lysosomal storage to cell growth arrest. FASEB J..

[27] Aureli M., Loberto N., Lanteri P., Chigorno V., Prinetti A., Sonnino S. (2011). Cell surface sphingolipid glycohydrolases in neuronal differentiation and aging in culture. J. Neurochem..

[28] Loberto N., Lunghi G., Schiumarini D., Samarani M., Chiricozzi E., Aureli M. (2018). Methods for Assay of Ganglioside Catabolic Enzymes. Methods Mol. Biol..

[29] Fazzari M., Audano M., Lunghi G., Di Biase E., Loberto N., Mauri L., Mitro N., Sonnino S., Chiricozzi E. (2020). The oligosaccharide portion of ganglioside GM1 regulates mitochondrial function in neuroblastoma cells. Glycoconj. J..

[30] Audano M., Pedretti S., Cermenati G., Brioschi E., Diaferia G.R., Ghisletti S., Cuomo A., Bonaldi T., Salerno F., Mora M. (2018). Zc3h10 is a novel mitochondrial regulator. EMBO Rep..

[31] Dookwah M., Wagner S.K., Ishihara M., Yu S.H., Ulrichs H., Kulik M.J., Zeltner N., Dalton S., Strauss K.A., Aoki K. (2023). Neural-specific alterations in glycosphingolipid biosynthesis and cell signaling associated with two human ganglioside GM3 synthase deficiency variants. Hum. Mol. Genet..

[32] Aureli M., Bassi R., Loberto N., Regis S., Prinetti A., Chigorno V., Aerts J.M., Boot R.G., Filocamo M., Sonnino S. (2012). Cell surface associated glycohydrolases in normal and Gaucher disease fibroblasts. J. Inherit. Metab. Dis..

[33] Mizushima N., Yoshimori T., Levine B. (2010). Methods in mammalian autophagy research. Cell.

[34] Yamashita T., Hashiramoto A., Haluzik M., Mizukami H., Beck S., Norton A., Kono M., Tsuji S., Daniotti J.L., Werth N. (2003). Enhanced insulin sensitivity in mice lacking ganglioside GM3. Proc. Natl. Acad. Sci. USA.

[35] Liu Y., Su Y., Wiznitzer M., Epifano O., Ladisch S. (2008). Ganglioside depletion and EGF responses of human GM3 synthase-deficient fibroblasts. Glycobiology.

[36] Russo D., Della Ragione F., Rizzo R., Sugiyama E., Scalabri F., Hori K., Capasso S., Sticco L., Fioriniello S., De Gregorio R. (2018). Glycosphingolipid metabolic reprogramming drives neural differentiation. EMBO J..

[37] Chatterjee S., Balram A., Li W. (2021). Convergence: Lactosylceramide-Centric Signaling Pathways Induce Inflammation, Oxidative Stress, and Other Phenotypic Outcomes. Int. J. Mol. Sci..

[38] Roseman S. (1970). The synthesis of complex carbohydrates by multiglycosyltransferase systems and their potential function in intercellular adhesion. Chem. Phys. Lipids.

[39] Preti A., Fiorilli A., Lombardo A., Caimi L., Tettamanti G. (1980). Occurrence of sialyltransferase activity in the synaptosomal membranes prepared from calf brain cortex. J. Neurochem..

[40] Chakraborty S., Doktorova M., Molugu T.R., Heberle F.A., Scott H.L., Dzikovski B., Nagao M., Stingaciu L.R., Standaert R.F., Barrera F.N. (2020). How cholesterol stiffens unsaturated lipid membranes. Proc. Natl. Acad. Sci. USA.

[41] Lunghi G., Carsana E.V., Loberto N., Cioccarelli L., Prioni S., Mauri L., Bassi R., Duga S., Straniero L., Asselta R. (2022). beta-Glucocerebrosidase Deficiency Activates an Aberrant Lysosome-Plasma Membrane Axis Responsible for the Onset of Neurodegeneration. Cells.

[42] Cooper A., Wraith J.E., Savage W.J., Thornley M., Noronha M.J. (1991). beta-mannosidase deficiency in a female infant with epileptic encephalopathy. J. Inherit. Metab. Dis..

[43] Varki A., Cummings R.D., Aebi M., Packer N.H., Seeberger P.H., Esko J.D., Stanley P., Hart G., Darvill A., Kinoshita T. (2015). Symbol Nomenclature for Graphical Representations of Glycans. Glycobiology.

[44] Settembre C., Di Malta C., Polito V.A., Garcia Arencibia M., Vetrini F., Erdin S., Erdin S.U., Huynh T., Medina D., Colella P. (2011). TFEB links autophagy to lysosomal biogenesis. Science.

[45] Bodas M., Min T., Vij N. (2015). Lactosylceramide-accumulation in lipid-rafts mediate aberrant-autophagy, inflammation and apoptosis in cigarette smoke induced emphysema. Apoptosis.

[46] Bharathi S.S., Zhang B.B., Paul E., Zhang Y., Schmidt A.V., Fowler B., Wu Y., Tiemeyer M., Inamori K.I., Inokuchi J.I. (2022). GM3 synthase deficiency increases brain glucose metabolism in mice. Mol. Genet. Metab..

[47] Garcia-Ruiz C., Colell A., Paris R., Fernandez-Checa J.C. (2000). Direct interaction of GD3 ganglioside with mitochondria generates reactive oxygen species followed by mitochondrial permeability transition, cytochrome c release, and caspase activation. FASEB J..

[48] Indellicato R., Parini R., Domenighini R., Malagolini N., Iascone M., Gasperini S., Masera N., dall’Olio F., Trinchera M. (2019). Total loss of GM3 synthase activity by a normally processed enzyme in a novel variant and in all ST3GAL5 variants reported to cause a distinct congenital disorder of glycosylation. Glycobiology.

[49] Inokuchi J.I., Go S., Suzuki A., Nakagawasai O., Odaira-Satoh T., Veillon L., Nitta T., McJarrow P., Kanoh H., Inamori K.I. (2024). Dietary gangliosides rescue GM3 synthase deficiency outcomes in mice accompanied by neurogenesis in the hippocampus. Front. Neurosci..

[50] Chester M.A. (1998). IUPAC-IUB Joint Commission on Biochemical Nomenclature (JCBN). Nomenclature of glycolipids--recommendations 1997. Eur. J. Biochem..

