# Supplementary material for: Metabolic and Structural Consequences of GM3 Synthase Deficiency: Insights from an HEK293-T Knockout Model
Source: Biomedicines. 2025 Apr 1;13(4):843. doi: 10.3390/biomedicines13040843 (PMC12024672; doi:10.3390/biomedicines13040843)
Supplement: Supplementary file 1 [file biomedicines-13-00843-s001.zip › biomedicines-3515221-supplementary.pdf]

biomedicines

MDPI

Supplementary Information

Metabolic and Structural Consequences of GM3 Synthase Deficiency: Insights from an HEK293-T Knockout Model

Elena Chiricozzi [1,*,†], Giulia Lunghi [1,†], Manuela Valsecchi [1], Emma Veronica Carsana [1], Rosaria Bassi [1], Erika Di Biase [2], Dorina Dobi [1], Maria Grazia Ciampa [1], Laura Mauri [1], Massimo Aureli [1], Kei-ichiro Inamori [3], Jin-ichi Inokuchi [4], Sandro Sonnino [1] and Maria Fazzari [1,*]

[1] Department of Medical Biotechnology and Translational Medicine, Università Degli Studi di Milano, 20054 Segrate, Italy; giulia.lunghi@unimi.it (G.L.); manuela.valsecchi@unimi.it (M.V.); emma_sn@hotmail.it (E.V.C.); rosaria.bassi@unimi.it (R.B.); dorina.dobi@unimi.it (D.D.); maria.ciampa@unimi.it (M.G.C.); laura.mauri@unimi.it (L.M.); massimo.aureli@unimi.it (M.A.); sandro.sonnino@unimi.it (S.S.)

[2] The Broad Institute of MIT and Harvard, Cambridge, MA 02142, USA; edibiase@broadinstitute.org

[3] Division of Glycopathology, Institute of Molecular Biomembrane and Glycobiology, Tohoku Medical and Pharmaceutical University, Sendai 981-8558, Japan; kinamori@tohoku-mpu.ac.jp

[4] Forefront Research Center, Graduate School of Science, Osaka University, Toyonaka 565-0871, Japan; inokuchi@chem.sci.osaka-u.ac.jp

* Correspondence: elena.chiricozzi@unimi.it (E.C.); maria.fazzari@unimi.it (M.F.)

† These authors equally contribute to this work.

Supplementary Figure S1

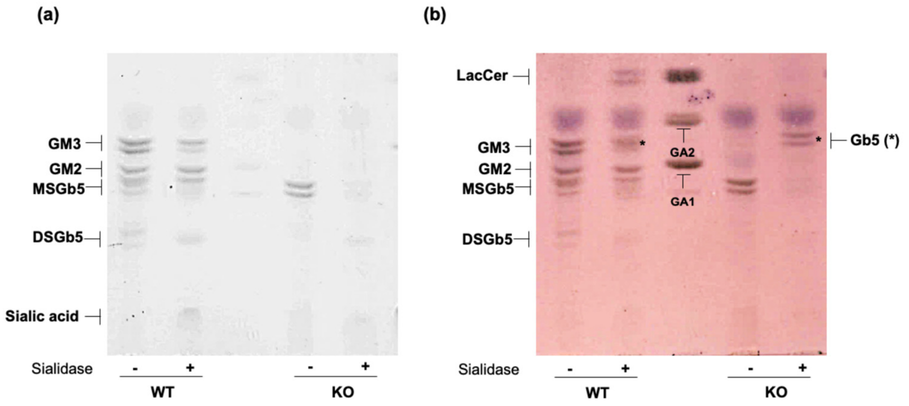

Figure S1. Identification of sialylated lipid pattern in WT and GM3S KO HEK293T cells. Repre-sentative HPTLC analysis of lysates obtained from WT and GM3S KO cells subjected (+) or not (-) to Vibrio cholerae sialidase treatment and visualized (**a**) by Ehrlich reagent and, subsequently, (**b**) by anisaldehyde reagent. By comparing a and b HPTLC plates and exploiting lipid standards that have been previously analyzed by mass spectrometry [24], it emerged that sialidase treatment leads to production of LacCer and sialic acid starting from GM3 and to the generation of Gb5 and sialic acid from MSGb5 and DSGb5 in WT lysates; no changes in GM2 expression level was found. On the other hand, MSGb5 and DSGb5 were converted to Gb5 and sialic acid in GM3S KO lysates upon sialidase addition. Additionally, no expression of GA1 and GA2 lipid species is observed in both cell cultures indicating that 0-series gangliosides are not produced in HEK293T cell line. Patterns are representative of those obtained in five indipendent experiments. * is used to indicate Gb5 in the image.

 https://doi.org/10.3390/biomedicines13040843
